# Development of a refined neonatal rabbit model of necrotizing enterocolitis with relatively longer survival

**DOI:** 10.3389/fphys.2025.1643482

**Published:** 2025-09-22

**Authors:** Qing-Qing Guo, Hao Lin, Zhi-Xin Wang, Jian-Bo Wen, Juan-Juan Yang, Shi-Yun Lu

**Affiliations:** ^1^ Department of Intensive Care Unit, The First Affiliated Hospital, Fujian Medical University, Fuzhou, Fujian, China; ^2^ Shengli Clinical Medical College, Fujian Medical University, Fuzhou, Fujian, China; ^3^ Department of Gastroenterology, Fuzhou University Affiliated Provincial Hospital, Fuzhou, Fujian, China; ^4^ Department of Gastroenterology, Afffliated PingXiang Hospital, Gannan Medical University, Pingxiang, Jiangxi, China; ^5^ College of Biological Science and Biotechnology, Fuzhou University, Fuzhou, Fujian, China

**Keywords:** animal model, intestinal tissue, necrotizing enterocolitis, New Zealand rabbits, protein expression

## Abstract

**Objective:**

This study aimed to develop an animal model that closely replicates the clinical features of necrotizing enterocolitis (NEC), thereby providing a supportive platform for ongoing NEC research.

**Methods:**

36 two-week-old neonatal suckling rabbits were randomly assigned to three groups: normal group (Group A, n = 12), traditional group (Group B, n = 12), and modified group (Group C, n = 12). Daily monitoring of general condition, post-modeling survival rate, and survival time was conducted in neonatal rabbits. Histopathological assessment of intestinal morphology was performed using hematoxylin and eosin staining, with scoring conducted to evaluate modeling success. Serum concentrations of C-reactive protein (CRP), tumor necrosis factor-alpha (TNF-α), interleukin-10 (IL-10), occludin, zonula occludens-1 (ZO-1), and zonulin were quantified using ELISA. Protein expression levels of occludin and ZO-1 in intestinal tissues were analyzed via Western blot.

**Results:**

Modified group exhibited significantly higher survival rates and prolonged survival time compared to traditional group (p < 0.05). Both model groups demonstrated elevated serum levels of CRP, TNF-α, IL-10, along with reduced concentrations of occluding, ZO-1 and zonulin when compared to normal group (p < 0.05). Similarly, intestinal expression levels of occludin and ZO-1 were significantly lower in both model groups relative to control (p < 0.05). However, no significant differences were observed between model groups in these parameters (p > 0.05).

**Conclusion:**

Our study successfully established a novel neonatal rabbit model of NEC with prolonged survival time by refining conventional modeling methodologies, thereby providing a new platform for investigating NEC pathogenesis and therapeutic interventions.

## 1 Introduction

Necrotizing enterocolitis (NEC) is a severe gastrointestinal disorder primarily affecting preterm neonates, with incidence rates inversely correlated with gestational age. It is characterized by an exaggerated inflammatory response accompanied by intestinal edema, hemorrhage, and necrosis, often resulting in intestinal perforation. (Neu and Walker, 2011). Common clinical manifestations include vomiting, abdominal distension, diarrhea, and hematochezia. (Sim et al., 2015). Although the underlying pathogenesis of NEC remains incompletely understood, it is widely accepted to be multifactorial. (Sanchez and Kadrofske, 2019). Contributing factors include prematurity, formula feeding, microbial colonization, intestinal inflammation, and hypoxia-ischemia. (Koike et al., 2020).

Currently conservative treatment options, such as probiotics, antibiotics, and supportive care, have shown limited efficacy. (Wang et al., 2019). In cases refractory to medical management, surgical resection of necrotic intestinal segments may be required. However, surgical intervention is associated with high mortality rates, reported to range from 40% to 50%, as well as increased risk of postoperative complications such as short bowel syndrome, neurodevelopmental delay, and sepsis, with an estimated incidence of 40%–70%. (Carr and Gadepalli, 2019). NEC imposes a substantial burden not only on affected neonates but also on families and healthcare systems due to its high morbidity and associated care costs. (Mowitz et al., 2018). Due to the absence of specific and effective therapeutic options, continued in-depth investigation into the pathophysiology and treatment of NEC remains essential.

The development of reliable and clinically relevant animal models is essential for exploring pathological mechanisms and for evaluating potential therapeutic strategies. Conventional animal models of NEC typically involve exposure to hypertonic formula, hypothermia, and cold stress over a period of 3–5 days. (Ravisankar et al., 2018; Sheng et al., 2015). However, several limitations reduce the translational value of these models. First, the timing of NEC onset in these animal models does not correspond with the typical onset window in human neonates, which generally occurs between 14 and 21 days of life. (Gan et al., 2019). Second, the survival duration in conventional models is short, often ranging from only 2–4 days, restricting the feasibility of extended observation and evaluation. (Ravisankar et al., 2018; Sheng et al., 2015; Roy et al., 2018). Third, simultaneous implementation of modeling and therapeutic interventions introduces confounding factors that compromise the validity of experimental outcomes. (Satoh et al., 2016; Li et al., 2017). Finally, the exclusive use of hypertonic formula for nutritional support during modeling does not reflect the complexity of clinical nutritional management in NEC. (Ravisankar et al., 2018; Roy et al., 2018).

In response to these limitations, the present study aimed to establish a modified NEC model in neonatal suckling rabbits that more accurately simulates clinical disease characteristics. Key modifications included enhancement of nutritional support through parenteral nutrition (PN) to prolong survival and facilitate extended observation periods. Two-week-old suckling rabbits were randomly assigned to three groups: a modified model group, a conventional model group, and a standard breastfeeding control group. NEC induction in both model groups involved hypertonic formula, hypothermia, and hypoxic stress, with the modified group additionally receiving PN. The control group was maintained on maternal breastfeeding. Daily monitoring was conducted to assess general health status. Pathological scores, modeling success rates, and survival outcomes were recorded on days 0, 4, 10, and 17. In addition, serum inflammatory markers and indicators of intestinal barrier integrity were evaluated to characterize systemic and localized effects of the induced NEC models.

## 2 Materials and methods

### 2.1 Experimental animals and groupings

A total of 39 two-week-old New Zealand white suckling rabbits (both sexes) were obtained from Songlian Experimental Animal Farm, Songjiang District, Shanghai (license number: SCXK [Shanghai] 2017-0008). Experimental grouping was conducted using a segmented randomized design. Based on body mass, the rabbits were stratified into four sections: Section 1 (160–175 g), Section 2 (176–190 g), Section 3 (191–205 g), and Section 4 (206–220 g). After excluding three animals prior to experimentation, 36 suckling rabbits were randomly allocated into three groups (n = 12 per group) as follows: Group A: (normal group): received breastfeeding only; Group B: (traditional group): subjected to a combination of homemade hypertonic formula, hypoxia, and hypothermia; Group C: (modified group): received PN in addition to the same modeling conditions as Group B. All experimental procedures involving animals were reviewed and approved by the Animal Ethics Committee of Pingxiang Hospital, affiliated with Gannan Medical University (Approval No: 2022243).

### 2.2 Main reagents and instruments

#### 2.2.1 Drugs and nutritional formulations

Abbott Plus Protein Powder (Abbott, United States), Gimlet Puppy Breast Milk Substitute (PetAg, United States), 20% fat emulsion, 5% compounded amino acids, water-soluble vitamins for injection, fat-soluble vitamins for injection, and Andamax (Huarui Pharmaceutical Co.).

#### 2.2.2 Primary reagents

Enzyme-linked immunosorbent assay (ELISA) kits for C-reactive protein (CRP), tumor necrosis factor-alpha (TNF-α), interleukin-10 (IL-10), and zonulin (Jianglai Biotechnology Co., Shanghai, China); ELISA kits for occludin and zonula occludens-1 (ZO-1) (Thermo Fisher Scientific, United States); primary antibodies for occludin and ZO-1 (Abcam, United Kingdom; ab216327 and ab96587, respectively); β-actin antibody (Immunoway Corporation, United States; YM3028); secondary antibodies for occludin and ZO-1 (Kangwei Century Biological Co., Beijing, China); and secondary antibody for β-actin (Solarbio, Beijing, China).

#### 2.2.3 Main instruments

CY-100 portable digital oxygen meter (Meicheng Electrochemical Analysis Instrument Factory, Jiande, Zhejiang Province); refrigerator maintained at 4 °C (Huamei Cold Chain Technology Co., Zhejiang, China); airtight anoxic chamber and custom-built incubator for neonatal rabbits.

### 2.3 Induction of animal models

Based on findings from preliminary trials in suckling rabbits, the protocol for inducing the conventional NEC model was refined as follows (Zani et al., 2008):

1. The concentration of Abbott Plus Protein Powder in the hypertonic formula was reduced from 200 g/L to 133 g/L.
2. The frequency of daily gavage was decreased to three times per day.
3. All rabbits were provided with maternal breast milk for 3 days prior to induction for adaptive feeding.

**FIGURE 1:**
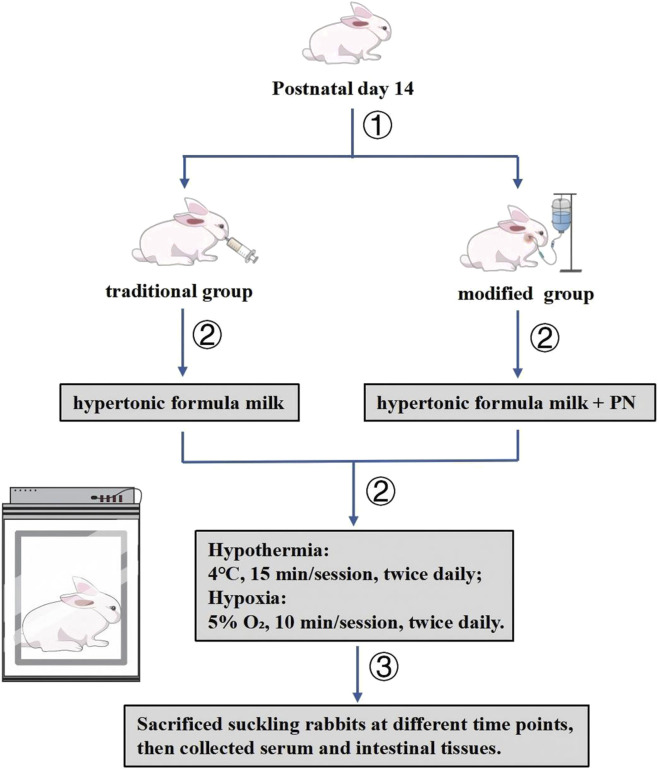
Establishment method of a modified suckling rabbit model of Necrotizing Enterocolitis with relatively longer survival.

In the modified group, NEC was induced using a combination of homemade hypertonic formula, hypothermia, and hypoxia. To enable prolonged survival for observation under sustained catabolic stress, PN was administered concurrently as the sole energy source throughout the induction phase. In the traditional group, NEC induction was achieved using the same protocol of hypertonic formula, hypothermia, and hypoxia, while receiving isovolumetric saline in place of PN from the outset of the experiment. The normal group received maternal breastfeeding only, without any additional interventions beyond equal-volume milk feedings. The induction phase lasted for 3 days in both model groups. From day four onwards, animals in the modified group were sustained with PN as the sole energy source, while those in the traditional group were provided with an isovolumetric isotonic formula (Figure 1). The normal group continued breastfeeding for the duration of the experimental period.

Induction procedures were conducted as follows:

1. Gavage feeding: A hypertonic formula prepared with 10 g Abbott Plus Protein Powder and 75 mL Gimlet milk substitute was administered at 15 mL/(kg·time), three times daily, with intervals of at least 4 h. The gavage volume was adjusted according to the animal’s daily weight.
2. Hypothermic and hypoxic stimulation: Hypothermia was induced by placing animals in a refrigerator maintained at 4 °C for 10 min following the first and third round of gavage each day. Subsequently, hypoxia was induced by placing the animals in a chamber filled with a gas mixture of 95% nitrogen and 5% oxygen at a flow rate of 10 L/min for 10 min. These interventions were applied twice daily (Figure 1).
3. PN: In the modified group, central venous access was established via the internal jugular vein 1 day prior to induction. PN was initiated on the day of modeling and maintained throughout the experiment. The PN formulation was adapted from previously published protocols, with a reduced glucose content to account for the energy derived from formula feeding during the induction phase (see Table 1 for formulation details). (Wu et al., 2007; Cai et al., 2013) During the initial three-day induction period, PN was administered at 4 mL/kg/h for approximately 12 h per day, resulting in a daily total of 50 mL/kg and 40 kcal/kg. From day four onwards, the infusion rate was increased to 5 mL/kg/h for over 16 h per day, totaling approximately 100 mL/kg and 80 kcal/kg daily.
4. Volume-matched intervention in the traditional group: During the induction phase, animals in this group received saline in volumes equivalent to those of PN. From day 4 onward, they were provided with an equivalent volume of isotonic formula for nutritional support.
5. Analgesia: To alleviate potential pain and distress associated with the procedures, all animals in the two intervention groups (traditional and modified) received subcutaneous injections of carprofen at a dose of 4 mg/kg once daily throughout the modeling period.
6. Housing conditions: Outside of hypothermic or hypoxic stimulation periods, all animals were housed in incubators maintained at a temperature of 30°C–32 °C and relative humidity of 60%–80%.

**TABLE 1:** Detailed formula of nutrient solution for parenteral nutrition.

Composition	Content (mL)	Energy (kcal)
20% Fat Emulsion	55	110
5% Compounded Amino Acids	160	32
10% dextrose injection	100	40
50% dextrose injection	56	112
10% Sodium Chloride Injection	4	-
10% Potassium Chloride Injection	3	-
10% Calcium gluconate	3	-
the water-soluble vitamins	5	-
the fat-soluble vitamins	5	-
Andamax	5	-
Total	396	294

### 2.4 General condition monitoring of suckling rabbits

The overall condition of the suckling rabbits was assessed every 6 h from the onset of the modeling phase. Observations included mental status, feeding behavior, activity levels, stool characteristics, abdominal distension, and signs of respiratory distress. Body weight was recorded daily at a consistent time point in the morning. Animals exhibiting moribund conditions were humanely euthanized in accordance with ethical guidelines to facilitate timely specimen collection.

### 2.5 Timing of specimen collection

Prior to the initiation of modeling, three suckling rabbits were euthanized to obtain baseline blood and small intestinal tissue samples. Subsequently, three animals from each group were euthanized at specified time points: Day 1, Week 1, and Week 2 following the modeling procedure (corresponding to experimental Days 4, 11, and 17, respectively). Blood and intestinal tissue samples were collected by trained personnel using aseptic technique. Samples were transported on ice, promptly aliquoted, and stored at −80 °C. In addition, specimens were collected immediately upon identification of moribund animals to preserve sample integrity.

### 2.6 Histopathological evaluation of the intestine

#### 2.6.1 Gross examination

Euthanized animals were positioned on a sterile dissection platform, and the abdominal cavity was opened following serum sample collection. The entire gastrointestinal tract, including the stomach and intestinal segments, was excised, rinsed with sterile saline in a Petri dish, and transferred to a clean tray for macroscopic examination. Gross pathological features of the intestines were documented and photographed.

#### 2.6.2 Light microscopic examination

Approximately 2–3 cm of distal ileum tissue (from a blinded intestinal section) was isolated and subjected to hematoxylin and eosin (H&E) staining. Histopathological scoring of the intestinal tissue was performed using the Nadler scale as follows (Nadler et al., 2000):

Score 0: Intact intestinal villi and epithelium with normal histological appearance.

Score 1: Mild separation and swelling in the submucosa or lamina propria.

Score 2: Moderate separation and swelling in the submucosa or lamina propria, with edema in submucosal or muscular layers.

Score 3: Severe separation and swelling in the submucosa or lamina propria, with marked edema in the submucosal or muscular layers, and localized villous loss.

Score 4: Complete villous loss with evidence of intestinal necrosis.

Histopathological evaluations were conducted by two independent investigators under blinded conditions. In cases of significant discrepancy, a third pathologist was consulted to reach a consensus. A final pathology score of ≥2 was defined as indicative of NEC and was used to determine the modeling success rate in each group. Representative pathological images were captured and archived.

### 2.7 Survival analysis and modeling success rate

Mean survival time and survival rates were calculated at different time points (prior to modeling, during induction, and following the modeling period), based on the survival duration of individual animals. The modeling success rates of the two model groups were determined using histopathological scores, enabling comparison of differences in modeling outcomes between the groups.

### 2.8 ELISA for serum inflammatory factors and intestinal mucosal injury markers

Whole blood samples were allowed to clot at room temperature for 2 h, followed by centrifugation at 4 °C for 20 min at 2000 rpm. The serum was subsequently collected and stored at −20 °C. Concentrations of CRP, TNF-α, IL-10, occludin, ZO-1, and zonulin were quantified using ELISA kits, according to the manufacturers’ instructions.

### 2.9 Western blot analysis of small intestinal mucosal injury markers

Total cytoplasmic proteins were extracted from small intestinal tissues stored at −80 °C and quantified using the bicinchoninic acid method. A 50 μg protein sample from each extract was subjected to sodium dodecyl sulfate-polyacrylamide gel electrophoresis, followed by transfer to a polyvinylidene fluoride membrane. Membranes were blocked with 5% skimmed milk prior to antibody incubation.

Primary antibodies were incubated overnight at 4 °C. After washing, membranes were incubated with secondary antibodies at room temperature for 1 h with gentle agitation. Protein expression levels of occludin and ZO-1 were detected using the ECL method. All procedures were carried out in strict accordance with the respective reagent protocols.

### 2.10 Statistical analysis

All statistical analyses were performed using SPSS software version 22.0 (SPSS Inc., Chicago, IL, United States). Quantitative data are presented as mean ± standard deviation (x̄ ± s). One way analysis of variance or non-parametric tests were employed for intergroup comparisons, while categorical data were analyzed using the chi-squared test. Survival analyses were conducted using the Kaplan-Meier method. A *p*-value of <0.05 was considered statistically significant.

## 3 Results

### 3.1 Alterations in the general status of suckling rabbits

#### 3.1.1 Changes in general condition

Suckling rabbits in the traditional group began exhibiting signs of lethargy, diminished reactivity, and reduced activity approximately 36 h after the initiation of the experiment. After 48 h, these rabbits developed profuse diarrhea, including instances of hematochezia, accompanied by a high mortality rate. In the modified group, signs of lethargy, decreased activity, abdominal bloating, and diarrhea were observed starting on the seventh day after modeling (i.e., the 10th day of the experiment). Most of these rabbits experienced diarrhea, bloody stools, and death between the eighth- and ninth-days following modeling (i.e., the 11th and 12th days of the experiment). Mortality data across the three groups at various time points are presented in Table 2.

**TABLE 2:** Mortality in the three groups of suckling rabbits at each time point.

Group	Experiment day 2		Experiment day 4		Experiment day 11		Experiment day 12	
	Deaths	Survival	Deaths	Survival	Deaths	Survival	Deaths	Survival
A	0	12	0	9	0	6	0	6
				(Sampling of 3)		(Sampling of 3)		
B	7	5	11	1	12	0	12	0
C	0	12	3	9	6	6	10	2

A: normal group; B: traditional group; C: modified group.

The numbers of dead suckling rabbits listed in the table above are all those that were on the verge of death or had died due to NEC., Three healthy suckling rabbits in the control group were executed on day 4 and day 11 of the experiment, which were not counted as dead because they were in good condition.

#### 3.1.2 Changes in body mass

**FIGURE 2:**
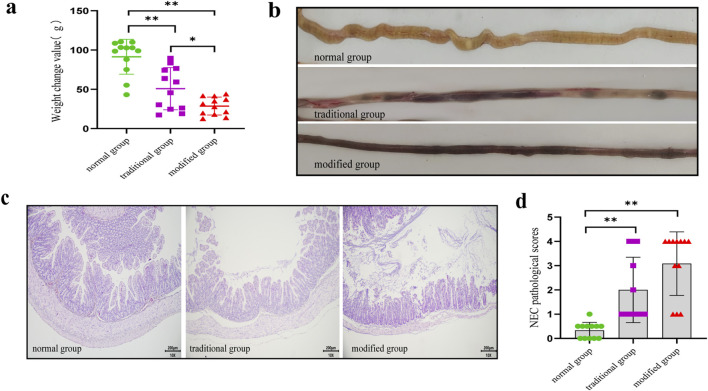
Alterations in general status and intestinal histopathological changes of suckling rabbits in each group. **(a)** Changes in body mass. **(b)** Macroscopic presentations of the intestine. **(c)** Comparison of pathological sections under light microscope: Sections were stained with hematoxylin and eosin (HE) using standard techniques and photographed at ×10 magnification. **(d)** NEC pathological scores. Note: *p < 0.05, **p < 0.001.

Body mass in the normal group increased significantly during the experimental period, whereas the model groups demonstrated significant decreases. Notably, both model groups demonstrated significantly different body mass changes compared to the control group (normal group vs. traditional group: 91.20 ± 22.11 g vs. 69.42 ± 43.67 g; normal group vs. modified group: 91.20 ± 22.11 g vs. 30.58 ± 12.49 g; *p* < 0.001) (Figure 2a). Additionally, body mass loss was significantly greater in the modified group compared to the traditional group (*p* < 0.05).

### 3.2 Intestinal histopathological changes

#### 3.2.1 Gross specimen observations

In the normal group, the intestines of suckling rabbits exhibited yellowish coloration with good elasticity and no observable signs of congestion or distention. In contrast, the intestines from both model groups demonstrated varying degrees of intestinal dilation, congestion, and discoloration, ranging to a purplish hue. No significant differences in gross intestinal pathology were identified between the two model groups (Figure 2b).

#### 3.2.2 Light microscopic observations

Histological analysis of intestinal tissue in the normal group revealed normal structural features, including intact epithelial linings and villi, orderly glandular architecture, and absence of mucosal, submucosal, or lamina propria congestion, edema, or separation. In contrast, both model groups exhibited extensive histopathological damage. Noted features included villous degeneration, glandular disorganization or loss, tissue fragmentation, necrosis, and detachment of epithelial structures. Edema and separation of the submucosa and lamina propria were also observed, along with thinning or rupture of the muscularis layer (Figure 2c).

#### 3.2.3 Pathological scores

Mean histopathological scores were 0.33 ± 0.49 for the normal group, 2.09 ± 0.66 for the traditional group, and 3.07 ± 1.36 for the modified group. Both model groups exhibited significantly higher scores compared to the normal group (*p* < 0.001), indicating more severe intestinal injury. Although the modified group demonstrated slightly higher mean scores than the traditional group, the difference was not statistically significant (*p* > 0.05) (Figure 2d).

### 3.3 Survival time, survival rate and modeling success rate of suckling rabbits

#### 3.3.1 Survival time

**FIGURE 3:**
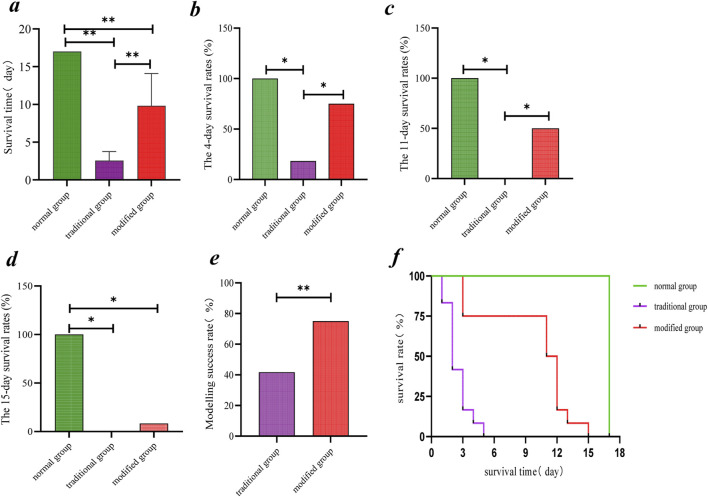
Comparison of survival time, survival rate and modeling success rate in suckling rabbits. **(a)** Survival time. **(b)** 4-day survival rate. **(c)** 11-day survival rate. **(d)** 15-day survival rate. **(e)** Modeling success rates among model groups. **(f)** Kaplan-Meier survival curve. Note: *p < 0.05, **p < 0.001.

The mean survival time in the normal group was 17.00 ± 0.00 days. In the traditional group, the mean survival time was significantly shorter, at 2.55 ± 1.21 days (*p* < 0.001 vs. normal). The modified group exhibited an intermediate survival duration, with a mean of 9.83 ± 4.26 days, which was also significantly shorter than the normal group (*p* < 0.001) but significantly longer than the traditional group (*p* < 0.001) (Figure 3a).

#### 3.3.2 Survival rates

1. At 4 days post-experiment, survival rates were 100% in the normal group, 75.0% in the modified group, and 8.3% in the traditional group. Both model groups exhibited significantly lower survival rates compared to the normal group; however, the difference between the modified group and the normal group was not statistically significant. The modified group had a significantly higher survival rate than the traditional group (*p* < 0.05) (Figure 3b).
2. By day 11, survival rates were 100% for the normal group, 50.0% for the modified group, and 0% for the traditional group. Both model groups had significantly reduced survival compared to the normal group (*p* < 0.05), and the survival rate in the modified group was significantly higher than that in the traditional group (*p* < 0.05) (Figure 3c).
3. At day 15, survival rates were 100% in the normal group, 8.3% in the modified group, and 0% in the traditional group. Both model groups had significantly lower survival rates than the normal group (*p* < 0.05), but no statistically significant difference was observed between the two model groups (Figure 3d).

#### 3.3.3 Modelling success rate

The modeling success rate was 41.7% in the traditional group and 75.0% in the modified group. Although the modified group exhibited a higher success rate, the difference was not statistically significant (*p* < 0.001) (Figure 3e).

#### 3.3.4 Survival curves

Survival curve comparisons indicated a significantly lower survival rate in the traditional group relative to both the normal and modified model groups (*p* < 0.001). Additionally, the modified group demonstrated a significantly lower survival rate than the normal group (*p* < 0.001) (Figure 3f).

### 3.4 ELISA for serum inflammatory markers: CRP, TNF-α, and IL-10

#### 3.4.1 CRP comparison

**FIGURE 4:**
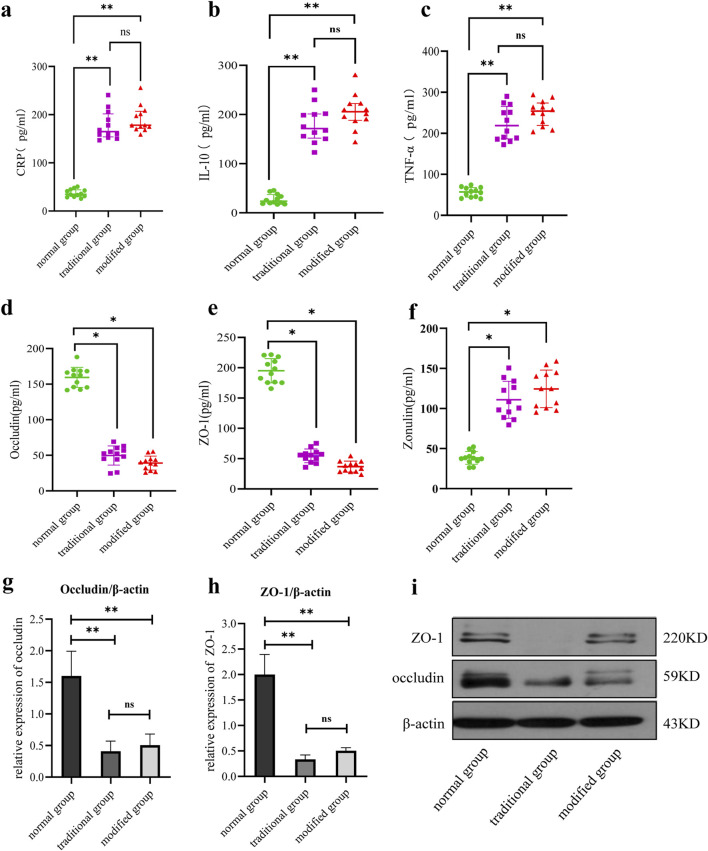
Comparison of serum inflammatory factors and intestinal mucosal damage markers among all groups. **(a)** Serum CRP level. **(b)** Serum IL-10 level. **(c)** Serum TNF-α levels. **(d)** Serum occludin level. **(e)** Serum ZO-1 level. **(F)** Serum zonulin protein level. **(g)** Intestinal occludin level. **(h)** Intestinal ZO-1 level. **(i)** Comparison of intestinal tight junction protein expression. Note: *p < 0.05, **p < 0.001.

Serum CRP levels were significantly elevated in both the traditional and modified groups compared to the normal group (*p* < 0.001). Although the CRP level in the modified group was slightly higher than in the traditional group, this difference did not reach statistical significance (*p* > 0.05) (Figure 4a).

#### 3.4.2 IL-10 comparison

IL-10 expression was significantly increased in both model groups relative to the normal group, with statistically significant differences observed (*p* < 0.001). While IL-10 levels were slightly higher in the modified group than in the traditional group, the difference was not statistically significant (*p* > 0.05) (Figure 4b).

#### 3.4.3 TNF-α comparison

Serum TNF-α levels were significantly elevated in both model groups compared to the normal group (*p* < 0.001). A slightly higher TNF-α concentration was observed in the modified group compared to the traditional group, but the difference lacked statistical significance (*p* > 0.05) (Figure 4c).

### 3.5 ELISA for serum occludin, ZO-1, and zonulin

#### 3.5.1 Occludin comparison

Occludin expression levels were significantly reduced in both model groups compared to the normal group (*p* < 0.001). Although the occludin level in the modified group was higher than that in the traditional group, the difference was not statistically significant (*p* > 0.05) (Figure 4d).

#### 3.5.2 ZO-1 comparison

ZO-1 expression levels were significantly decreased in both model groups relative to the normal group, with statistical significance (*p* < 0.001). The modified group demonstrated a slightly higher ZO-1 level compared to the traditional group, but this difference did not reach statistical significance (*p* > 0.05) (Figure 4e).

#### 3.5.3 Zonulin protein comparison

Zonulin expression levels were significantly elevated in both model groups compared to the control group (*p* < 0.05). The zonulin level in the modified model group was slightly higher than that in the general model group, although this difference was not statistically significant (*p* > 0.05) (Figure 4f).

### 3.6 Western blot assays for occludin and ZO-1

#### 3.6.1 Occludin comparison

Occludin expression was significantly reduced in both model groups compared to the normal group, with no significant difference observed between the two model groups (Figures 4g,i).

#### 3.6.2 ZO-1 comparison

Similarly, ZO-1 expression was significantly decreased in both model groups relative to the normal group, with no significant difference observed between the two model groups (Figures 4h,i).

## 4 Discussion

The findings of this study indicate that, relative to the conventional modeling approach, the modified NEC modeling method applied to suckling rabbits confers distinct advantages, including prolonged survival time and disease characteristics that more closely resemble the pathogenesis observed in clinical NEC. Consequently, the modified method represents a superior approach for generating animal models of NEC.

Due to the absence of specific clinical symptoms and physical signs, histopathological changes in the intestine remain the definitive standard for clinical diagnosis of NEC. (Shao, 2019). These changes primarily encompass neutrophil infiltration in the intestinal wall, disruption of villous structures, submucosal edema, and hemorrhage. (Papillon et al., 2013). Furthermore, pathological evaluation constitutes the gold standard for confirming successful NEC model establishment. (Nadler et al., 2000). In the present study, both model groups demonstrated marked disruption of intestinal tissue architecture, including villous destruction and submucosal congestion and edema, as observed under light microscopy. These histopathological alterations conformed to the NADLER scoring criteria for NEC diagnosis and were consistent with pathological findings reported in pediatric NEC cases.

In addition to microscopic pathology, gross morphological changes observed in intestines affected by NEC in pediatric patients include dilatation, thinning of the intestinal wall, congestion with a purple-black discoloration, necrosis, and perforation. (Ballanc et al., 1990). The gross intestinal alterations identified in the model groups in this study predominantly involved diminished elasticity, severe dilatation, congestion, and necrosis, closely mirroring those seen in clinical NEC.

The maintenance of intestinal mucosal barrier integrity relies on tight junction proteins such as ZO-1 and occludin, which are critical for epithelial barrier function. (Slifer and Blikslager, 2020). Zonulin, a human protein that modulates intestinal permeability through interactions with tight junction proteins, serves as a valuable biomarker for intestinal mucosal injury. (Barbaro et al., 2020).

Ravisankar et al. reported that disruption of tight junction proteins compromises gut barrier function, permitting translocation of microbial products and toxins into systemic circulation, thereby contributing to the pathogenesis of diseases including NEC and sepsis. (Ravisankar et al., 2018). Similarly, Bein et al. observed that expressions of occludin and ZO-1 are significantly reduced in neonates with NEC compared to healthy controls, implicating increased intestinal permeability and gut tissue necrosis in affected patients. (Bein et al., 2018). In alignment with these observations, the current study found significantly elevated serum zonulin levels along with decreased serum and intestinal expressions of tight junction proteins in both model groups relative to controls. These results collectively indicate pronounced intestinal mucosal injury, necrosis, and increased permeability in the modeled suckling rabbits.

It is now recognized that NEC is associated with increased localized expression of various inflammatory factors, including interleukin-6 (IL-6), interleukin-8 (IL-8), IL-10, transforming growth factor, and TNF, which serve as nonspecific markers for NEC. (Cho et al., 2016). In the present study, serum levels of inflammatory factors—CRP, TNF-α, and IL-10—were significantly elevated in the model groups compared to controls, indirectly confirming the successful establishment of the NEC model.

The construction of reliable animal models constitutes the foundation of experimental research; consequently, the validity of related investigations depends on whether these models accurately replicate clinical disease characteristics such as age of onset, survival time, and the capacity to simulate clinical treatment processes. Clinically, most neonates with NEC develop the disease between 11 and 24 days of age. (Gan et al., 2019). However, conventional NEC models often utilize neonatal rats that are sacrificed after only 2–3 days of induction, which does not align with the typical age of disease onset in human neonates. (Ravisankar et al., 2018; Sheng et al., 2015).

In contrast, this study employed 2-week-old suckling rabbits, with disease induction occurring after 3 days, thereby more closely reflecting the clinical timing of NEC onset. The primary clinical manifestations of NEC in neonates include hematochezia, abdominal distension, vomiting, and hypoactive bowel sounds, which correspond to the symptoms observed in the experimental model groups. (Shao, 2019). The current clinical mortality rate for neonates with NEC within 5 days of admission is approximately 12%, and survival rates for Bell stage I and II patients can exceed 80% with conservative medical management. (Shao, 2019; Luig et al., 2005). A significant increase in both survival time and survival rate in the modified model group was demonstrated in this study, thereby approximating the survival outcomes observed in clinical pediatric populations.

Many existing experimental NEC studies initiate treatment interventions concurrently with disease induction, a practice that may compromise experimental accuracy. (Satoh et al., 2016; Li et al., 2017). In contrast, the extended survival time and increased survival rate observed in the modified model group allowed for the administration of interventions only after the modeling phase, minimizing potential confounding effects and enhancing the precision of experimental results.

Clinically, neonates with NEC often require intensive maintenance therapy, including empirical antibiotic treatment, gastrointestinal decompression, PN, fluid resuscitation, correction of hematological and metabolic imbalances, and support for circulatory and respiratory functions. (Knell et al., 2019). While experimental constraints preclude full replication of these complex clinical treatment conditions, this study enhanced the energy supply to suckling rabbits through simulated clinical PN, which contributed to correcting water-electrolyte imbalances and thus more closely approximated the clinical management of NEC.

This improved experimental protocol may have broader applicability beyond the construction of a prolonged NEC model. Animal models of severe acute pancreatitis, severe ulcerative colitis, and severe cholangitis are often associated with high mortality rates due to extensive fluid loss, elevated energy demands, and complications such as infectious shock. (Antoniou et al., 2012; Xu et al., 2010; Hu et al., 2014). Enhancing energy supply and facilitating fluid resuscitation through PN via jugular vein catheterization during the modeling period may prolong survival, reduce mortality, and provide an extended window for subsequent experimental interventions. Additionally, the potential application of this modified modeling approach to other inflammatory bowel disease models, including ulcerative colitis and Crohn’s disease, warrants further investigation and represents a future research focus of the present study team.

Nevertheless, this study has several limitations. First, the relatively small sample size in this study may have influenced the reliability of the results. Second, while the provision of supportive care via the central venous access is the most plausible explanation for the prolonged survival, the precise physiological and molecular mechanisms behind this improvement remain unexplored in this study. Finally, it is important to acknowledge that all current NEC models, including the modified one presented here, are developed based on incomplete understanding of the disease’s exact etiology and pathophysiology. Therefore, future studies are necessary not only to validate these findings with larger sample sizes but also to utilize this model to investigate the specific mechanisms of survival improvement and to continue refining modeling techniques as our understanding of NEC deepens.

In conclusion, beyond prolonged survival, our modified NEC model offers several key advantages: (1) Enhanced clinical relevance by more closely mimicking the human disease course; (2) A prolonged therapeutic window that facilitates the investigation of disease mechanisms and the evaluation of potential treatments; (3) Elimination of confounding factors by temporally separating the disease induction phase from subsequent experimental interventions, thereby increasing the accuracy of experimental outcomes; and (4) The versatility of the central venous access, which not only provides essential nutritional and fluid support but also serves as a platform for administering various therapeutics (e.g., antibiotics, anticoagulants), making the experimental process more aligned with clinical practice. Notably, this ‘life line’ platform is applicable beyond NEC research to other severe disease models such as severe acute pancreatitis and sepsis.

## Data Availability

The original contributions presented in the study are included in the article/supplementary material, further inquiries can be directed to the corresponding authors.

## Abbreviations

NEC, necrotizing enterocolitis; PN, parenteral nutrition; HE, hematoxylin and eosin; CRP, C-reactive protein; TNF-α, Tumor Necrosis Factor -α; IL-10, Interleukin-10; ZO-1, Zonula Occludens-1; BCA, Bicinchoninic Acid; PVDF, Polyvinylidene fluoride; ECL, Enhanced Chemiluminescence; ANOVA, analysis of variance; IL-6, Interleukin-6; IL-8, Interleukin-8; TGF, Transforming Growth Factor; TNF, Tumor Necrosis Factor.
